# Review of "Surgical disorders of the peripheral nerves" (2^nd ^Edition) by Rolfe Birch

**DOI:** 10.1186/1749-7221-7-3

**Published:** 2012-04-13

**Authors:** Gerhard Blaauw

**Affiliations:** 1 Department of Neurosurgery, University Hospital Maastricht, The Netherlands

## Book Review

The London school of treatment and study of peripheral nerve injury was the heir of the Medical Research Council's Peripheral Nerve Injury Unit in Oxford during the Second World War. H.J. Seddon was appointed as surgeon in charge of the Unit during the wartime and he was able to continue at the Royal National Orthopaedic Hospital and the Institute of Orthopaedics in London after the end of the war. After Seddon's retirement distinguished colleagues (Brooks, Bonney, and others) developed and extended the work of all these men. Rolfe Birch has extended the work of these leaders and he presents us with this book which bears the same title as Seddon's classic monograph. Birch dedicated his book to George Bonney.

The work has been almost entirely rewritten with much greater emphasis upon the causes and manifestations of injuries to nerves, particularly iatrogenic injuries.

The book contains 14 chapters, covering basic information: anatomy, microscopic structure, reactions to injury, regeneration and recovery, clinical aspects of nerve injury and clinical neurophysiology, detailed information on operating on peripheral nerves and an important chapter covering aspects of rehabilitation. Special comprehensive attention is applied to brachial plexus injuries, both adult lesions but more in particular to birth lesions. This edition is richly illustrated, a great number in color. Each chapter is followed by numerous references.

Thus a new landmark is available for those who are interested in the back ground of brachial plexus and nerve surgery. This book reflects the personal ideas of a great teacher and a gifted surgeon richly illustrated by results from surgeons who worked in the past in this subject.

